# The Effect of Transcranial Direct Current Stimulation on Relapse, Anxiety, and Depression in Patients With Opioid Dependence Under Methadone Maintenance Treatment: A Pilot Study

**DOI:** 10.3389/fphar.2020.00401

**Published:** 2020-04-03

**Authors:** Mohammad Sadeghi Bimorgh, Abdollah Omidi, Fatemeh Sadat Ghoreishi, Amir Rezaei Ardani, Amir Ghaderi, Hamid Reza Banafshe

**Affiliations:** ^1^Department of Addiction Studies, School of Medical, Kashan University of Medical Sciences, Kashan, Iran; ^2^Department of Clinical Psychology, School of Medicine, Kashan University of Medical Science, Kashan, Iran; ^3^Clinical Research Development Unit, Matini/Kargarnejad Hospital, Kashan University of Medical Sciences, Kashan, Iran; ^4^Psychiatry and Behavioral Sciences Research Center, Mashhad University of Medical Sciences, Mashhad, Iran; ^5^Physiology Research Center, Institute for Basic Sciences, Kashan University of Medical Sciences, Kashan, Iran; ^6^Department of Pharmacology, School of Medicine, Kashan University of Medical Sciences, Kashan, Iran

**Keywords:** transcranial direct current stimulation, opioid dependence, relapse, depression, anxiety, stress

## Abstract

**Background and Objective:**

Patients under methadone maintenance therapy (MMT) are susceptible to several complications including mental disturbances and risk of relapse. The present study was designed to evaluate the effects of tDCS on relapse, depression, and anxiety of opioid-dependent patients under methadone maintenance treatment (MMT).

**Methods:**

It was a randomized-clinical trial that conducted among 27 male patients referred to the outpatient addiction clinic of Ibn-e-Sina psychiatric hospital in Mashhad from July 2018 to May 2019. Participants were allocated to two treatment groups including intervention and sham groups. The intervention group received seven sessions of tDCS, in the F3 (cathode) and F4 (anode) areas of the brain, each one lasts 20 min, in two consecutive weeks. Depression, anxiety, and stress scale-21 (DASS-21) were measured before, during, and after the intervention in patients under MMT. Relapse on the morphine, cannabis, and methamphetamine was screened by urine dipstick tests of morphine, cannabis, and methamphetamine.

**Results:**

Depression, anxiety, and stress of participants were significantly reduced in the intervention group compared with the control after the seventh session of tDCS (P < 0.001, P=0.01, and P=0.01, respectively). In addition, the relapse rate showed no significant changes between the two groups (P=0.33).

**Conclusion:**

Overall, our study demonstrated that depression, anxiety, and stress of participants were significantly reduced after the seventh session of tDCS, but did not affect on the relapse rate. Therefore, it can be applied as a safe and effective technique to relieve mental disorder among receiving MMT.

**Clinical Trial Registration:**

http://www.irct.ir, identifier IRCT20180604039979N1.

## Introduction

Addiction is a complex disease that is manifested by compulsive substance use despite harmful consequences (Bromley, 1992). Nowadays, a large number of people diagnosed having drug use disorders and suffered from physical, psychological, cultural, economic, and social consequences of using substances (Lievens et al., 2017). The World Health Organization's annual report estimates that there are about 200 million opiate addicts in the world (0.6 to 0.8% of the general population), while Iran has nearly three times higher prevalence compared with the mean prevalence of the world. Eastern Iran shares a border with Afghanistan where the majority of opium in the world is produced. Iran is the major route for drug transport into Europe (Amirabadizadeh et al., 2018; Soroosh et al., 2019). The most commonly used opioid in Iran is opium (82%), followed by opium ashes (28%), methadone for non-medical usages (16.6%), heroin and heroin/cracked (16%), and morphine (2.6%). Apart from opioids, the common substances that are illegally used in Iran include alcohol with a prevalence of 2% within the past 12 months, cannabis with a prevalence of 1%, and methamphetamine with a prevalence of 0.5%. There are around 5000 outpatient buprenorphine or MMT clinics for the sole purpose of the treatment of opioid dependency in Iran and covered about 500,000 people for treatment (Eskandarieh et al., 2014; Amin-Esmaeili et al., 2016; Ghaderi et al., 2017).

According to the National Institute on Drug Abuse, between 40% and 60% of people recovering from drug addiction will relapse (Saitz, 2007). The strong desire to re-experience the effects of the substances after reaching the abstinence, known as craving, leads to the high relapse rate in patients (Ahmadpanah et al., 2013). Studies have shown that 20 to 90% of addicts who receive treatment will relapse in the next 6 months (Sotodeh Asl et al., 2013). Therefore, treatment of the addiction usually targets the craving in order to decrease the relapse rate. In some cases, mental disorders such as anxiety, depression, or schizophrenia precede addiction; in other cases, drug use disorder may trigger or worsen those mental health conditions, particularly in people with specific vulnerabilities (Langas et al., 2011). So, any treatment protocol for drug addiction usually requires several courses of treatment and various treatment modalities (Scott et al., 2005).

Transcranial Direct Current Stimulation (tDCS) is one of the techniques of brain stimulation which involves the use of a weak direct electric current (e.g., 1 to 2 mA) through two or more electrodes, usually an anode and a cathode, on the scalp (Talimkhani et al., 2019). This stimulation acts as a non-invasive, inexpensive, and safe method for altering the resting potential of cortical neural cells. Anodal stimulation leads to an increase of cortical excitability, whereas cathodal stimulation reduces cortical excitability. tDCS has few temporary side effects, including burning sensation and itching under the electrodes, headache, and fatigue (Fitzgerald et al., 2014; Fertonani et al., 2015). However, these side effects are reported to be mild and transient (Brunoni et al., 2014b; Cosmo et al., 2015). According to brain imaging findings, the dorsolateral prefrontal cortex (DLPFC) has an essential role in the pathophysiology of drug craving and emotional distress in patients suffering from addiction (da Silva et al., 2013). DLPFC has been implicated in spontaneous and cue-elicited cravings. It is involved in different cognitive tasks such as decision making, inhibitory control, and attentional bias, too (Luigjes et al., 2019). Therefore, DLPFC could be a target for stimulation in order to alleviate the craving and relapse rate of patients with drug dependence disorder. However, stimulation of the DLPFC may affect the reward system *via* efferent pathways from ventral tegmental area to the nucleus accumbens (Luigjes et al., 2019). As these pathways are involve in the hedonic responses of the brain to the environmental stimuli (Heshmati and Russo, 2015). Stimulating DLPFC could attenuate negative emotional states (e.g. depression and anxiety) of patients.

These lines of evidence emphasize the importance of tDCS on relapse and mental health, suggesting tDCS may have favorable effects in patients under MMT programs. In addition, it may potential impact with nursing care, functional activities, rehabilitation potential, and functional recovery. To our knowledge, data from studies investigating the effects of tDCS on promote mental health and relapse in MMT patients are limited. Therefore, this study was aimed to evaluate the effect of tDCS on relapse, depression, and anxiety among patients who underwent MMT.

## Materials and Methods

### Trial Design and Participants

**Diagram 1:**
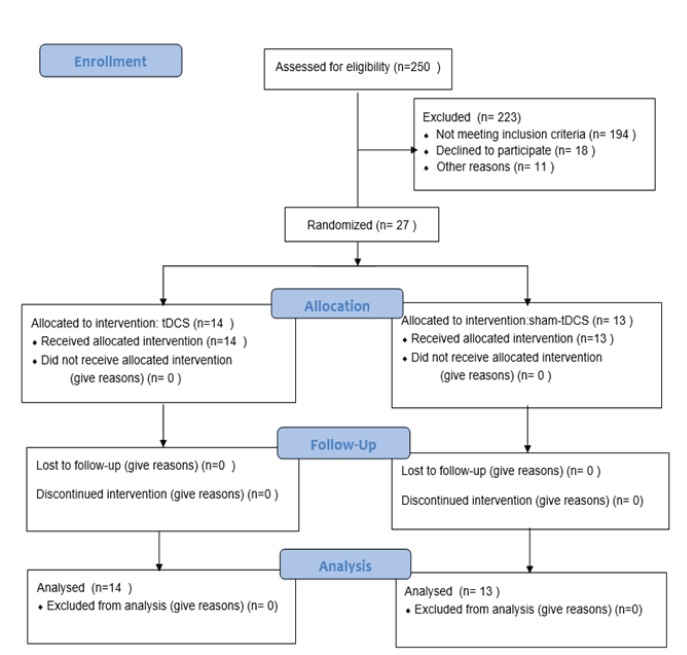
Summary of patient flow diagram.

This randomized, double-blinded, sham-controlled clinical trial, registered in the Iranian clinical trials website at (http://www.irct.ir: **IRCT20180604039979N1**), followed the Declaration of Helsinki guideline, informed consent was given by all patients. This trial was conducted among 27 participants undergoing MMT, who referred to the Ibn-e-Sina Hospital in Mashhad, Iran, from July 2018 to May 2019. Twenty-seven patients were selected through convenient sampling method based on the criteria. Then, they were divided into the experimental (n=14) and the sham groups (n=13) (**Diagram 1**). In term of ethical considerations, the researchers provided the needed information to all patients such as process of treatment, probably side effects of tDCS (such as sense of mild burning or headache) and duration of treatment. Then, they ask patients to write consent form. All patients received tDCS in 7 sessions (every other day). The study was approved by the ethics committee of Kashan University of medical sciences.

### Inclusion Criteria

Participants included into the study based on the following criteria: receiving MMT as the only treatment protocol and for less than 1 year, evaluated by the drug abuse section of the Structured Clinical Interview for DSM-IV, aged between 18 and 60 years, educational attainment of higher or equal to mid-high school (necessary for completing the questionnaires), not having diabetes and specific neurological diseases including the history of head trauma and seizure, not having any metal objects or prosthesis near the site of brain stimulation, not having cerebral shunt or cardiac pacemaker.

### Exclusion Criteria

The exclusion criteria of the study were the patient's unwillingness to continue participation, any undesirable side effects attributed to tDCS, or patients' refusal to continue the methadone maintenance treatment.

### Randomization and Blinding

Randomization was performed with blanced blocked randomizaton and random numbers generated by computer software (Stat Trek software) which choose the random numbers. Randomization and allocation concealment were conducted by the researchers and participants and were carried out by a trained staff at the clinic. Subjects were randomly allocated into two groups to receive tDCS or sham tDCS. Also, the participants and researcher were blinded, but the research assistant was not blinded. Research assistant regulate device on real or sham mode in the intervention or the sham groups.

### Outcomes of Interest

Relapse rate was considered as the primary outcomes of interest. Depression and anxiety were considered as the secondary outcomes.

### Research Instruments

1. Researcher-made background profile form: This form was used to collect the demographic variables, the daily dosage of methadone consumed by participants and the duration of MMT of each participant.
2. Urine dipstick tests for the detection of morphine, cannabis, and methamphetamine: According to the American Society for Addiction Medicine (ASAM), drug testing should be used “to discourage nonmedical drug use and diversion of controlled substances, to encourage appropriate entry into addiction treatment, to identify an early relapse, and to improve outcomes of addiction treatment (Hadland and Levy, 2016). Urine dipstick drug tests are practical tools for assessing the relapse in primary care (McDonell et al., 2016). They are lateral-flow test strips which are analyzed the urine sample based on the immunochromatography technique. In the present study, we used Rojan dipsticks (Rojan Azma Company, Tehran, Iran), which are designed for the detection of morphine, cannabis, and methamphetamine, because of the high relapse rate of these substances. Using dipsticks are very simple, and the results obtained in about 5 min. The results are reported as positive, negative, and invalid according to the number of color bands appear in the control and the test region of the strips (Zare et al., 2016). The Positive result is interpreted as having a relapse, the negative result is interpreted as to have no relapse, and the invalid results need to repeat the urine test.
3. Depression, Anxiety, and Stress Scale-21 (DASS-21): The original version of this scale has 42 questions. However, we used the short version, called DASS-21. DASS-21 contains 21 items, seven questions measure depression, seven items measure anxiety, and the rest of them are for measuring stress. The depression sub-scale measures dysphoria, devaluation of life, hopelessness, self-deprecation, anhedonia, lack of interest, and inertia. The anxiety sub-scale assesses autonomic arousal, situational anxiety, muscle tension, and subjective experience of anxiety; while the stress sub-scale measures the levels of chronic arousal, difficulty relaxing and being easily upset, irritable, and impatient. Each respondent asked to indicate the presence of any symptoms over the last week. Each question is scaled on the 4-points Likert scale from 0 to 3. To calculate the final score of DASS-21, the final score of each sub-scale (depression, anxiety, and stress) should be multiplied by two (Lovibond, 1995). The Persian translation of DASS-21 is reported to be valid by (Sahebi et al., 2005; Babazadeh et al., 2016). Internal consistency of the DASS-21 subscales with Cronbach's alphas for depression (0.91), anxiety (0.87), and stress (0.90) respectively reported (Yazdi et al., 2019). In the present study, we considered the anxiety and stress sub-scales as different aspects of anxious affect. So, depression subscale score of the DASS-21 is interpreted as the level of depression, while anxiety and stress subscales scores are interpreted as the level of anxiety of the participants.

### Research Process

Transcranial Direct Current Stimulation (tDCS) usually involves the use of a weak and direct electric current (e.g. 1 to 2 mA) through two or more electrodes which are placed on the scalp (Talimkhani et al., 2019). The connection of two electrodes with different poles (usually an anode and a cathode) in different parts of the skull surface, leads to the stimulation of the lower neurons. Anodal stimulation induces an increase of cortical excitability, whereas cathodal stimulation decreases cortical excitability (Brunoni et al., 2012). Safety of the use of tDCS has been reported in human adults (Brunoni et al., 2014b; Cosmo et al., 2015).

**Figure 1:**
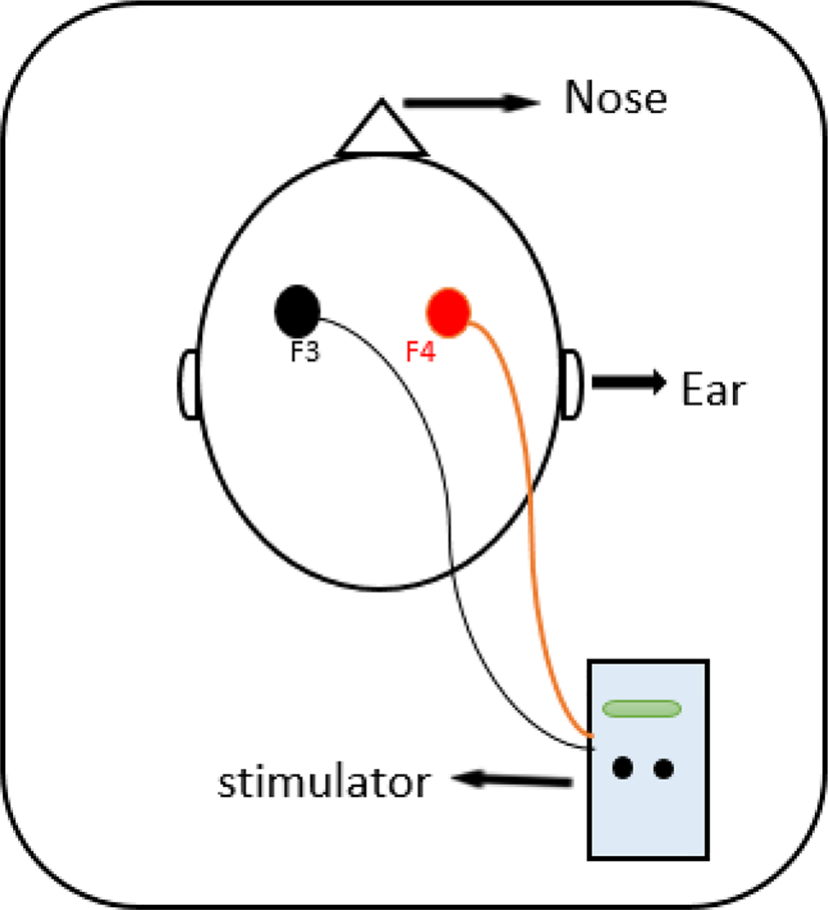
The anode was placed on the right DLPFC (F4) and cathode was placed on the left DLPFC (F3) according to the 10–20 international system for EEG.

In the present study, the intervention group received 7 sessions of tDCS, once a day, every other day with the intensity of 2 mA for 20 min. For anodal stimulation, the anode electrode was placed over right DLPFC (F4) and for cathodal stimulation, the cathode electrode was centered over left DLPFC (F3) based on international 10–20 EEG system (Batista et al., 2015) (**Figure 1**). Direct current was transferred *via* two electrodes coated in saline-soaked sponges with 35-cm^2^ size and delivered by a battery-driven stimulator (Oasis Pro, Mind Alive, Canada). For the sham group, the same methods were used, and the electrodes were placed in the same areas. However, the device was turned up to 2 mA for only 30 s to produce the sense of initial irritation, then slowly ramped-down to 0 mA over the period of 1 min, and finally turned off, while the electrodes stayed on the scalp up to 20 min.

In the first session, all participants were asked to fill out DASS-21 and screened for any relapses on morphine, amphetamine or cannabis by urinary dipstick before receiving tDCS or sham. Participants were asked to complete DASS-21 after the fourth the seventh sessions, while the same urinary screening test was conducted at the end of seventh session, too.

### Sample Size

Regarding the relapse rate as the main outcome, no study was found to calculate sample size. Therefore, this is a pilot study. Considering the obtained results of relapse rate in this study (7.1% and 23.1% for intervention and sham groups), the studies power was calculated 20% (P-value=0.05). It was obtained 15 patients for each group. However, considering the possibility of sample loss, we interred 30 participants in the study, but before randomization, three subjects declined to participation. Eventually, 27 patients terminated the study (14 in the intervention group and 13 in the sham group).

### Statistical Analysis

The Kolmogorov-Smirnov test was done to determine the normality of data. We used the Chi-square test, Independent sample *t*-test, repeated measures ANOVA, Fisher's exact test, and Mann-Whitney test to analyze the data, using SPSS-25. P-values <0.05 were considered statistically significant.

## Results

In the present study, we evaluated the effects of 7 sessions of tDCS on the depression, anxiety, stress, and relapse. No side effects were reported following tDCS sessions in patients under MMT. So, we did not have any excluded participant after the randomization.

Mean age, age of first illicit opioids use, marital status, education, job, and psychiatric comorbidity, use of other drugs, daily methadone dosage, and duration of MMT were not significantly different between the intervention and sham groups (**Table 1**).

**Table 1:** Comparison the background variables between the intervention and sham groups at the beginning of the study.^1^

Background Variables		Group		P
		Intervention	Sham	
Age (y)		37.36 ± 7.63	36.00 ± 5.69	0.69^*^
Age of first illicit opioids use (y)		21.42 ± 5.8	22.23 ± 4.04	0.68^*^
Daily methadone dosage (mg)		50.8 ± 17.4	52.0 ± 20.9	0.69^*^
Duration of MMT (months)		8.93 ± 2.46	8.31 ± 2.78	0.54^**^
Marital status (%)	Single	3 (21.4)	3 (23.1)	0.86^***^
	Married	10 (71.4)	8 (61.5)	
	Divorced	1 (7.2)	2 (15.4)	
Education (%)	Intermediate	10 (71.4)	10 (76.9)	0.74^***^
	Diploma	4 (28.6)	3 (23.1)	
Job (%)	Unemployed	9 (64.3)	4 (30.8)	0.09^***^
	Employed	1 (7.1)	0 (0)	
	Others	4 (28.6)	9 (69.2)	
Psychiatric comorbidity (%)	None	12 (85.7)	11 (84.6)	0.51^***^
	Mood disorder	1 (7.1)	0 (0)	
	Others disorders	1 (7.1)	2 (15.4)	
Use of other drugs (%)	None	12 (85.7)	12 (92.3)	0.22^***^
	Benzodiazepine	2 (14.3)	0 (0)	
	Antidepressants	0 (0)	1 (7.7)	

^1^Data are mean ± SD for quantitative variables and frequency (%) for qualitative variables.

*Mann-Whitney.

**Obtained from Independent t-test.

***Obtained from Chi-Square.

Comparison showed no significant differences about the relapse rate between the intervention and sham groups (P < 0.999 and P=0.33, respectively) (**Table 2**). Although no significant differences were found between the two groups in the mean score of depression, anxiety, and stress before and during the intervention, at the end of the study, significant differences were appeared between the two groups in all three variables (P < 0.001, P=0.01, and P=0.01, respectively).

**Table 2:** Comparison of relapse rate, mean depression, anxiety and stress scores between the intervention and sham groups before and after intervention.

Variables			Group		P
			Intervention Number	Sham Number	
**Relapse rate**	Before intervention	Negative	11(78.6)	10(76.9)	< 0.999^*^
		Positive	3(21.4)	3(23.1)	
	After intervention	Negative	13(92.9)	10(76.9)	0.33^*^
		Positive	1(7.1)	3(23.1)	
**Depression**	Before intervention		30. 71 ± 7.75	27.85 ± 8.46	0.37^**^
	During intervention (after the 4th session)		23.29 ± 7.04	28.31 ± 8.60	0.11^**^
	After intervention		17.57 ± 4.45	25.85 ± 7.09	< 0.001^***^
**Anxiety**	Before intervention		27.00 ± 8.25	25.38 ± 9.36	0.64^**^
	During intervention		22.29 ± 6.07	25.08 ± 8.31	0.33^**^
	After intervention		16.86 ± 4.20	23.23 ± 7.68	0.01^***^
**Stress**	Before intervention		33.00 ± 8.07	29.08 ± 8.23	0.14^***^
	During intervention		26.29 ± 7.23	27.69 ± 8.90	0.66^**^
	After intervention		17.28 ± 4.28	25.85 ± 8.38	0.01^***^

*Fischer's exact test.

**Independent t-test.

***Mann-Whitney.

**Figure 2:**
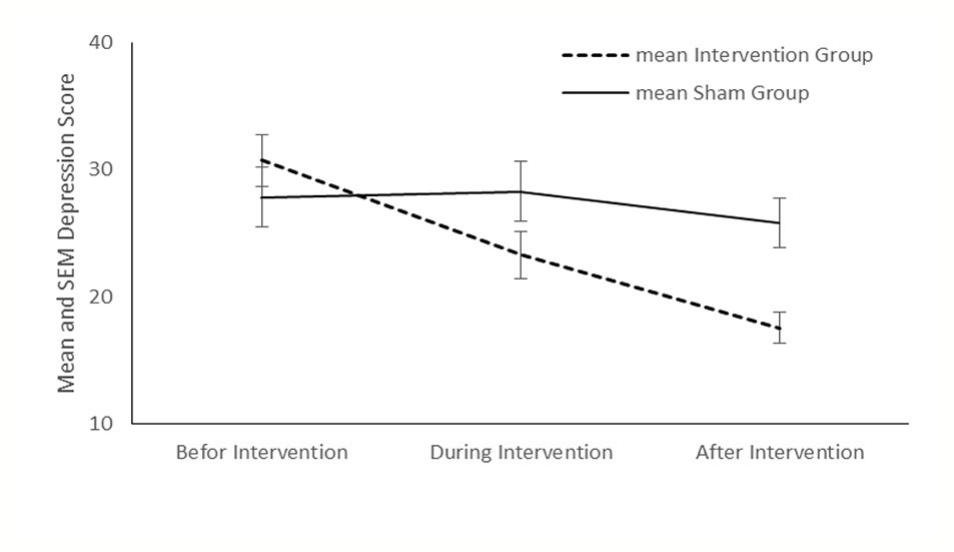
Mean depression score and in the intervention and sham groups at each time point.

**Figure 3:**
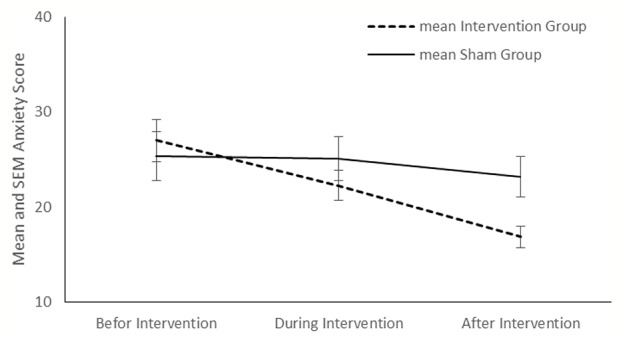
Mean anxiety score and in the intervention and sham groups at each time point.

**Figure 4:**
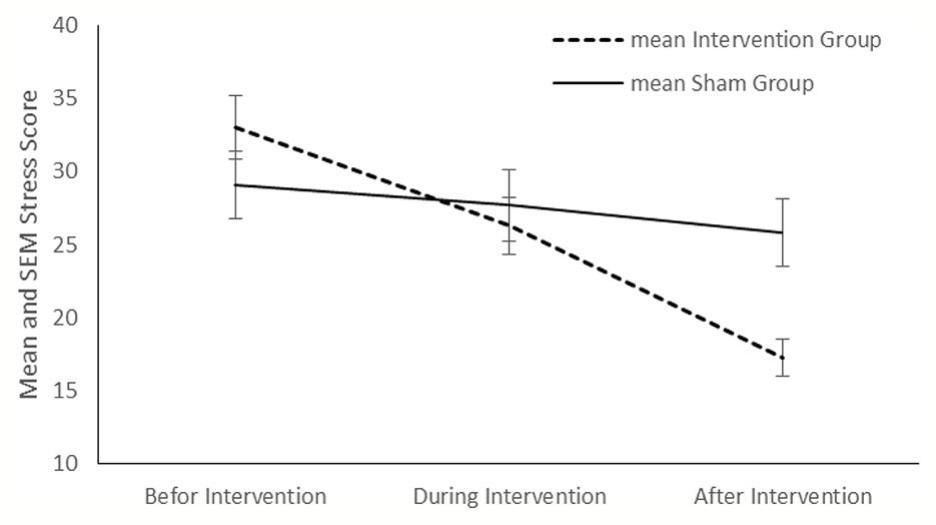
Mean stress score in the intervention and sham groups at each time point.

Compared with the sham group, tDCS resulted in a significant improvement in depression (P < 0.001), anxiety (P < 0.001) and stress (P < 0.001) in the intervention group (**Figures 2**–**4**). The *post hoc* comparison between the intervention and sham patients showed a significant group × treatment interaction on depression (F (2.50) =10.71; p < 0.001), anxiety (F (2.50) 6.48; P=0.003) and stress (F (2.50) 14.64; P < 0.001). Repeated measures depression as dependent variables produced a significant overall *F*-statistic for interaction effect of time and group; this finding indicated that the change in the scores on during the treatment was significantly different in tow groups.

## Discussion

The findings of the present study indicated that 7 sessions of tDCS did not conclude the significant differences between the two groups in relapse. Also, finding showed a significant difference in depression, anxiety, and stress rate between the intervention and sham groups at the end of the study. Although MMT is presented to reduce craving, relapse, and physical complaints of opiate users after drug discontinuation, some patients continue to experience serious and severe problems during the first years of abstinence, including depression, anxiety, and frequent relapses (Dobson and MOHAMMAD, 2006). In several studies, electrical stimulation was used as a left anodal/right cathodal and right anodal/left cathodal to reduce anxiety and depression (Brunoni et al., 2014a; Taremian et al., 2019). However, we apply the anode electrode on right DLPFC (F4) and the cathode electrode on the left DLPFC (F3). All participants tolerated the procedure and did report no side effects except for the redness of the stimulation site, which was resolved after a few minutes.

### Effects on Relapse

This study indicated that, compared with the sham groups, tDCS for 7 sessions in patients with opioid dependence under methadone maintenance treatment had no significant differences on the relapse rate. Researchers have performed different studies on the efficacy of the tDCS on the craving of patients with substance dependence disorders (Boggio et al., 2008; Sharifi-Fardshad et al., 2018; Garg et al., 2019; Martinotti et al., 2019). Garg et al. reported that the effect size of repeated sessions of tDCS targeted at prefrontal cortex (left anodal/right cathodal) on the craving reduction in patients with opioid dependence disorder was moderate. They could not show any significant difference in self-reported craving between the intervention and control groups (Garg et al., 2019). In another study, Taremian et al. examined the effect of tDCS at the dorsolateral prefrontal cortex (right anodal/left cathodal) on the craving to opium in a group of participants with opium use disorder enrolled in MMT program. The study showed that the active tDCS significantly reduced craving (Taremian et al., 2019). Martinotti et al. applied the same protocol (right anodal/left cathodal) on participants with substance use disorder or gambling disorder in which they reported a significant reduction of craving in the intervention group (Martinotti et al., 2019). Sharifi-Fardshad et al. compared the effects of active tDCS on the DLPFC with the right anodal and left anodal protocols with the control group and reported that right anodal active tDCS could reduce the craving in former heroin users under MMT (Sharifi-Fardshad et al., 2018). However, Wang et al. examined a different tDCS treatment protocol on a group of heroin-addicted participants. While they targeted the bilateral frontal-parietal-temporal circuitry and placed the cathodes over T3 and T4 and anodes over O2 and O1 (based on 10/20 EEG system), significant lower craving scores were reported after active treatment (Wang et al., 2016).

Although these studies showed promising effects of tDCS in decreasing the craving of patients with opioid dependence disorder, they may not be helpful enough in clinical practice. Assessment of craving in all the above-mentioned studies are based on self-reports questionnaires or scales. They did not measure clinical outcomes, and the literature suffered from the lack of data on the parameters such as relapses (Batista et al., 2015).We could consider the lapse/relapse as the clinical consequence of the craving; therefore, assessment of the relapse rate could be a good indicator of the clinical outcome of applying the tDCS on the craving.

In our study, applying the right anodal active tDCS, which has more evidence in reducing craving in different studies, could not significantly reduce the relapse rate in patients with opioid dependence disorder enrolled MMT program (Klauss et al., 2018; Taremian et al., 2019). However, after seventh session of tDCS, the intervention group had lower relapse rate compared to the sham group. Lack of significant efficacy of tDCS in our study could be due to the effect of MMT program on the relapse rate of participants. MMT could reduce the craving for opioids in opioid-dependent patients. Therefore, it could interfere with the efficacy of the tDCS. Other possible reasons of limited effect of tDCS could be the limited sessions of therapy and the limited number of participants of the study. All in all, right anodal/left cathodal protocol of tDCS seems to have little effects in reducing the relapse rate of patients enrolled MMT programs in non-laboratory settings.

### Effects on Depression and Anxiety

The current study demonstrated that tDCS for 7 sessions among patients with opioid dependence under methadone maintenance treatment significantly improved the depression, anxiety, and stress score compared with the sham subjects. Complaining of depressed mood is prevalent between patients suffering from opioid dependence disorder, even after replacing their illegal opioids with methadone. Half of the patients undergoing methadone maintenance treatment suffer from depression (Cosmo et al., 2015). Although depressed feelings after the first weeks of drug discontinuation could be self-limited, independent depression needs proper therapeutic management (Nunes and Levin, 2008). According to cerebral imaging, the dorsolateral prefrontal cortex (DLPFC) has an important role in the pathophysiology of mood disorders (Cosmo et al., 2015). Recently, tDCS has been recommended for treatment of depression with the anode over the left DLPFC (Bennabi and Haffen, 2018).

According to our study, there was no significant difference between the intervention and sham groups in the mean score of depression before and during the treatment. However, after the 7th session of tDCS, the mean score of depression in the intervention group was significantly lower than the sham group. While we applied a different stimulation site (the right DLPFC for anode electrode instead of the usual recommended stimulation site for treatment of depression), the significant therapeutic effect of tDCS on the depression score of participants was an interesting finding. Taremian and his colleagues applied the tDCS in a group of opioid-dependent individuals with MMT and reported a significant reduction in the self-reported craving, anxiety, and depression of the patients (Taremian et al., 2019). It seems that tDCS could affect the comorbid depression in patients under MMT, although it is not an early outcome. Comorbid depression in substance-dependent individuals has different explanations, including elevated risk of vulnerability for the second disease caused by the primary disease, common risk factors between disorders, using substances as self-medication of depression, residual depression from interpersonal or social consequences of addiction, delayed recovery caused by the comorbid disorder, and substance-induced depressive episodes caused by direct biological changes of addiction (Davis et al., 2008). As depression in opioid-dependent individuals is not a single disorder with a unique etiology, we suggest that the right anodal tDCS could have a therapeutic effect on some types of comorbid depression.

Anxiety is a well-known comorbidity of addiction (Cohn et al., 2011). While feeling anxious is related to the over-activity of the noradrenergic system, the core mechanism of action in opioid withdrawal syndrome is related to the noradrenaline release in the brain, too (Aston-Jones and Kalivas, 2008). Therefore, it is usual for opioid-dependent individuals to find themselves, feeling restless, anxious, and worried. Addressing anxiety has an important role in the management of addiction.

Anxious mood accompanies different psychological and somatic symptoms, including autonomic hyperactivity, restlessness, irritability, muscular twitching, etc. (Association, 2000). The anxiety and the stress sub-scales of DASS-21 assess these symptoms; therefore, we used these subscales to quantify anxiety score of participants. We found out that there was no significant difference between the intervention and sham groups in the mean scores of anxiety and stress sub-scales before and during the treatment. However, the intervention group showed a significant decline in anxiety and stress sub-scales compared to the sham group after the treatment.

Few studies have reported the effect of tDCS on the anxiety disorders and fewer in patients with substance dependence disorders (Hampstead et al., 2016; Kar and Sarkar, 2016). Some researchers have proposed the placement of cathode electrode on the right prefrontal cortex and anode electrode on the left prefrontal cortex (Stein et al., 2020). The hypothesis is that the cathodal stimulation may reduce cortical excitability in anxiety disorders, which accompany cortical excitability. Nevertheless, in a randomized sham-controlled trial of applying tDCS in patients with cocaine dependence, Batista et al. placed the anode electrode on the right DLPFC (similar to our study) and reported a significant decrease in the sense of anxiety in patients receiving tDCS in comparison to the sham group (Batista et al., 2015). Based on our findings, applying tDCS could reduce the anxiety symptoms in patients under MMT, though the outcome is achieved after seven consecutive sessions. The efficacy of tDCS on the anxiety symptoms in the Batista et al. and our study may be due to the overall improvement of the sense of health and well-being of the patients (Batista et al., 2015). However, more studies should target anxiety symptoms in patients with substance use disorders.

## Limitations

This study should be viewed in light of its limitations. Specifically, given it's a pilot study sample size, caution is advised before generalizing the results. Also, low duration of intervention that this is associated with illustrate lower effects. In addition, we were not able to investigate the withdrawal syndrome, chronic pain, and cognitive functions. Thus, its performance is suggested in next studies. Also, tDCS is a new method of brain stimulation. Therefore, most patients especially women were not familiar with it and refuse to participate in the study. In addition, the number of participants of the study was limited. Furthermore, the operator who conducted tDCS was not blind about the assigned patients (intervention and sham groups).

## Conclusions

The findings suggested the beneficial effect of tDCS to improve depression, anxiety, and stress in the experimental group compared to the sham subjects. This suggests that tDCS may confer advantageous therapeutic potential for patients under MMT programs. Further research is needed in other participants and for longer periods to determine the efficacy of tDCS.

## Data Availability Statement

The datasets generated for this study are available on request to the corresponding author.

## Ethics Statement

All procedures performed in studies involving human participants were in accordance with the ethical standards of the institutional and national research committee and with the 1964 Helsinki declaration and its later amendments. The patients/participants provided their written informed consent to participate in this study.

## Author Contributions

MS and HB contributed in design, conception, and statistical analysis. MS, AO, FG, AR, AG, and HB contributed in data collection and manuscript drafting.

## Funding

The present study was supported by a grant from the Vice-chancellor for Research, KAUMS, Iran.

## Conflict of Interest

The authors declare that the research was conducted in the absence of any commercial or financial relationships that could be construed as a potential conflict of interest.
